# Supplementary material for: A political economy of the tobacco supply chain in an Eastern Mediterranean country: The case of Lebanon
Source: PLoS One. 2025 Mar 31;20(3):e0320050. doi: 10.1371/journal.pone.0320050 (PMC11957285; doi:10.1371/journal.pone.0320050)
Supplement: S1 File — (DOCX) [file pone.0320050.s001.docx]

# **Supporting information**

Semi-structured interview guide (Key informants)

**Interview Guide Questions (Tobacco Industry)**

**The American University of Beirut**

Thank you for agreeing to speak to me today. I have some questions for you about the tobacco supply chain in Lebanon, the competitive structure of the tobacco industry, and the regulatory framework, particularly taxation, within the context of the protracted economic crisis in the country.

**Tobacco Supply Chain: Challenges, Dynamics, and Market Insights**

1. Can you provide an overview of the tobacco supply chain in Lebanon, including its historical development, key players, and major products?

a. Probe on the main components from cultivation to distribution

b. Probe on the main challenges and opportunities faced by the tobacco industry in Lebanon? How do they impact the supply chain?

c. Probe on different chain components

d. Probe on how the economic crisis have reshaped the industry (yes or no and how)

2. Can you discuss the relationships and dynamics between different stakeholders in the tobacco industry supply chain, such as farmers, manufacturers, distributors, and retailers? How do they interact and collaborate?

3. What are the key factors influencing the competitiveness of the tobacco industry in Lebanon? Are there any unique characteristics or advantages that differentiate it from other markets?

4. Can you provide insights into the local and international market demand for Lebanese tobacco products? How does this demand shape the supply chain and market strategies?

5. Are there any emerging trends or innovations within the tobacco industry in Lebanon that are affecting the supply chain? How are these trends being addressed by industry stakeholders?

**Regulations and Policies Governing the Tobacco Industry in Lebanon:**

6. How much do you think tobacco marketing and tobacco advertisements, promotion and sponsorship (TAPS) influence the smoking rate and smoking among young people?

7. Are there any specific regulations or policies governing the tobacco industry in Lebanon? How do these regulations affect the supply chain and its operations?

8. Can you please provide a brief description of the tax structure of the various tobacco products? Do you see any potential for an increase in tax revenue generation in this industry?

9. In your opinion, what are some potential policy measures that could enhance the regulation of the tobacco industry?

Semi-structured interview guide (Key informants)

| **Demographic Information:** Age of respondent |
| --- |
| Job and affiliated organization |
| Education level and expertise/ specialty |
| Time and date of interview |
| Location of the interview |
| Name of interviewer |
