# Supplementary material for: A political economy of the tobacco supply chain in an Eastern Mediterranean country: The case of Lebanon
Source: PLoS One. 2025 Mar 31;20(3):e0320050. doi: 10.1371/journal.pone.0320050 (PMC11957285; doi:10.1371/journal.pone.0320050)
Supplement: Attachment — Submitted filename: Responses to Reviewers RR PONE (Round 2).pdf [file pone.0320050.s004.pdf]

Responses to the Editor's and Reviewer's Comments for Manuscript PONE-D-24-14365

A Political Economy of the Tobacco Supply Chain in an Eastern Mediterranean Country: The Case of Lebanon

By Ali Abboud, Ali Chalak, Joanne Haddad, Mariam Radwan

Dear Professor Fabrizio Ferretti,

We sincerely appreciate your handling of our submission at PLOS ONE and thank you for the opportunity to revise our manuscript.

After carefully considering the feedback, we have prepared a fully responsive revision. We agreed with all the comments and have amended the paper accordingly. We hope that the revised version meets the editorial requirements and adequately addresses the concerns of both the academic editor and reviewers. Once again, we believe this process has significantly improved the substance and presentation of our analysis.

Throughout the document, we use boxes to highlight the reviewer comments, with our responses in plain text. Unless stated otherwise, all page, table, and figure references correspond to the revised version of the manuscript.

Unlike the first round of revisions, we have not included a summary of the major changes. Instead, we directly provide a detailed, point-by-point response to the reviewers' suggestions.

Ali Abboud, Ali Chalak, Joanne Haddad, Mariam Radwan

Journal Requirements

Please review your reference list to ensure that it is complete and correct. If you have cited papers that have been retracted, please include the rationale for doing so in the manuscript text, or remove these references and replace them with relevant current references. Any changes to the reference list should be mentioned in the rebuttal letter that accompanies your revised manuscript. If you need to cite a retracted article, indicate the article's retracted status in the References list and also include a citation and full reference for the retraction notice.

Response: We have carefully reviewed our reference list to ensure its completeness and accuracy. To verify that none of the cited papers have been retracted, we cross-checked them against the Retraction Watch database. Based on our review, we confirm that none of the references in our manuscript have been retracted.

Additional Editor Comments

Some statements have been presented throughout the manuscript without the addition of references or primary literature to support them, and it is unclear whether the information was obtained through the interviews or from other sources:

Response: We appreciate this feedback and have carefully reviewed the manuscript to ensure that all statements are properly supported by references or clearly attributed to the interviews. Where necessary, we have added citations to relevant primary literature to substantiate the claims. Additionally, we have explicitly clarified instances where information was derived from the interviews to ensure transparency.

"In practice, however, there is only minimal interaction and active oversight, which results in the Regie being an independently administered monopoly operating with very limited oversight by any elected or governmental body"

Reference not provided

Response: We thank the editor for highlighting the need for a citation. We have statements from both the Regie representative and the Ministry of Finance (MOF) representative supporting this claim, and we have amended the manuscript accordingly to reflect this.

"The Regie has the exclusive right to provide licenses to farmers and wholesalers, to purchase locally produced tobacco leaves from Lebanese farmers, to purchase and import raw tobacco material, to regulate and set tobacco products standards, as well as to manufacture and distribute tobacco products"

Reference not provided

Response: This information was mentioned in the interview with the Regie representative when he introduced the Regie. However, it is common knowledge and is also available on the Regie's website (https://www.regie.com.lb/Article/1/who-we-are/en). We have added a new footnote (#2) to the revised version of the manuscript to indicate this.

"According to a researcher involved in tobacco monopolies and taxation policies research, it is estimated that 5 to 6 billion dollars were transferred to the treasury between 1990 and 2020"

Reference not provided. Alternatively, a clarification from the authors of whether this researcher was part of the interviews.

Response: We thank the editor for raising this concern. We have revised the text to provide the necessary support for this claim.

We now write *"Based on an interview extract with a researcher involved in tobacco monopolies and taxation policies, it is estimated that 5 to 6 billion dollars were transferred to the treasury between 1990 and 2020. These numbers align with the data provided by the Regie in its financial overview, which is reported in Fig 1."*

"The Regie runs a subsidy program for tobacco farmers. The subsidy comes in the form of a negotiated price that is 60 to 100 percent higher than the global market price for tobacco leaves. Between 1994 and 2021, the subsidy cost was 1.032 billion USD."

Reference not provided.

Response: We thank the editor for this comment. We would like to clarify how we arrived at this statement. The total subsidy cost of 1.032 billion USD between 1994 and 2021 is based on data provided by the Regie in its financial overview, which is presented in Fig 1 of the manuscript. The claim that the subsidy price is 60 to 100 percent higher than the global market price was inferred from interviews with both the Regie representative and the Farmers Union representative. They noted that in 2023, the global market price for tobacco leaves was $3 per kg, while the Regie paid an average of $5 per kg, supporting the inference that the subsidy is 60 to 100 percent higher than the global price.

We have amended the manuscript to provide more clarity to the reader on how we arrived at this statement.

"The Regie has expanded its operations by manufacturing international brands since 2016."

Reference not provided

Response: This statement is based on an extract from an interview with the Regie representative. We have amended the manuscript to indicate so.

"More than a decade after the passage of the law, there has been a noticeable regression in its implementation, especially when it comes to enforcement of smoking bans in closed public spaces."

Reference not provided

Response: This statement is based on an extract from an interview with an anti-tobacco civil society activist. We have amended the manuscript to indicate so.

We now write *"One of the interviewees from civil society, who was involved in the effort to introduce this law, noted that more than a decade after its passage, there has been a noticeable regression in its implementation, particularly regarding the enforcement of smoking bans in closed public spaces. Nonetheless, the interviewee noted that, despite this setback, there are some lasting effects of Law No. 174, most notably the ban on advertisements."*

"This is evident from the significant smuggling activity in the direction of Syria. The Regie's willingness to satisfy this market demand is evident from their rapid increase in cigarette production in recent years."

This statement seems to be more of an author's opinion rather than supported by primary literature.

Response: Indeed**,** you are right, this is based on suggestive evidence. Several interviewees mentioned an increase in tobacco smuggling to Syria. The Regie representative did not deny this but stated something along the lines of, *"We produce, and maybe some of it goes to Syria, but we cannot control that."*

We now modify the manuscript to adjust the statement to make it less conclusive and more suggestive.

We now write *"A second source of this contradiction stems from the Regie de facto operating as a supplier of tobacco (mostly cigarettes) to a larger regional market, not just the Lebanese market. This is particularly evident given indications of significant smuggling activity toward Syria and the Regie's increased cigarette production in recent years, which may suggest a response to this market demand."*

Authors’ Response to Reviewer #2

Why does the study have strengths and limitations captured together in the same section? Isn’t the strengths part of the contributions of the study? I suggest that the strengths should be captured in the contribution and the section on limitation and future research direction must stand alone, as a component of the conclusion

Response: We thank the reviewer for this suggestion. We have revised the conclusion section to separate the strengths and limitations, rather than having them combined in the same section. Specifically, we now highlight both the theoretical and practical contributions of the study, in addition to its strengths. We have also created a standalone subsection dedicated to the study’s limitations and future research directions. We hope that our revisions adequately address the comments we received from you on this matter in both rounds of revisions.

All modifications have been made in track changes mode.

What is the limitation of the lack big tobacco firm participation on the results of the study? This must come out well

Response: We thank the reviewer for raising this concern. We have amended the sub-section on the limitations of the study to further discuss the limitation arising from the lack of participation from big tobacco firms. The subsection now reads as follows:

“The study has several limitations, including potential challenges in generalizing its findings to countries with significantly different supply chain structures in the tobacco industry. Additionally, relying solely on qualitative data may limit the depth of quantitative analysis needed to fully assess the economic impacts of proposed policy changes. Another limitation is the lack of participation from major tobacco firms in the study. On one hand, this is a limitation in terms of the data, as it leaves one side of the picture unexplored. On the other hand, given that this study focuses on the political economy of tobacco, the refusal of big tobacco firms to participate in the interviews may suggest a reluctance to be transparent, possibly indicating an interest in withholding information about the extent of their benefits from the current status quo.

There is also a possibility of bias in the data collected, given the strong political ties of the tobacco industry in Lebanon which may have influenced the responses of certain stakeholders, potentially introducing bias. Moreover, the feasibility and practicality of implementing the suggested policy interventions, especially within Lebanon's complex political and economic landscape, may pose significant hurdles that need careful consideration.”

The manuscript needs some proof reading, some parts do not read well.

Response: Thank you for your comment. We appreciate your feedback and have carefully proofread the manuscript to improve clarity and readability. We have made revisions to ensure that all sections flow smoothly and are easier to follow. Please let us know if there are any specific areas that you would like us to address further.
