# Supplementary material for: A political economy of the tobacco supply chain in an Eastern Mediterranean country: The case of Lebanon
Source: PLoS One. 2025 Mar 31;20(3):e0320050. doi: 10.1371/journal.pone.0320050 (PMC11957285; doi:10.1371/journal.pone.0320050)
Supplement: Attachment — Submitted filename: Responses to Reviewers RR PONE.docx [file pone.0320050.s003.docx]

# Responses to Reviewers’ Comments for Manuscript PONE-D-24-14365

# A Political Economy of the Tobacco Supply Chain in an Eastern Mediterranean Country: The Case of Lebanon

# By Ali Abboud, Ali Chalak, Joanne Haddad, Mariam Radwan

Dear Professor Muhammad Hashim,

We are very grateful to you as editor and to the anonymous reviewers for their comments, which were thoughtful, fair, and very helpful. We believe we have put together a fully responsive revision. In general, we agreed with the vast majority of comments and amended the paper in the directions suggested. We hope you will agree that the substance and packaging of the analysis has been considerably improved by the process of review and revision.

Throughout, we use boxes to identify the comments from the reviewer and plain text

capture our responses. Except where made explicit, page, table and figure numbers refer

to those in the new version.

We start by providing a summary of the major changes, followed by a point-by-point

response to the issues and suggestions raised by the referees.

Sincerely,

Ali Abboud, Ali Chalak, Joanne Haddad, Mariam Radwan

# Major Changes

## 1. Justifying Methodological Choices

To address the recurrent comment from both anonymous reviewers regarding the rationale behind our chosen methodology and the justification of each technique, we have made significant revisions to the manuscript. Specifically, we have edited the methodology section to thoroughly justify our methodological choices and explain the rationale behind each technique used.

### Summary of Changes:

1. We have provided a detailed explanation for using qualitative methods, supported by relevant academic literature and tobacco-related studies.
2. We have included a rationale for employing different techniques such as purposive sampling, induct
3. ive analysis approach, and thematic analysis, referencing scholarly sources to strengthen our justification.
4. We have referred to the suggested qualitative papers and have provided more in-depth description of the analysis process. Each step of the analysis is now mentioned in the methodology section to ensure more rigor and transparency of our results.

A detailed overview of these changes is provided in our response to the reviewers.

## 2. Clarifying the Research Gap, Objectives, and Contributions

To address one major comment raised by anonymous reviewer #2 regarding the need to better justify the study's relevance, clearly define the research question, and distinctly state the contributions of the study in comparison to previous research, we have made significant changes to the manuscript to address these concerns.

Specifically, we have:

1. We provided a comprehensive justification for the study's relevance, supported by a thorough review of the existing literature. In our discussion, we highlight how this study complements and adds to the existing body of work by addressing two significant issues in the Lebanese context: the existence of a profit-driven monopoly that conflicts with public policymakers' broader goals, including public revenue, health, and environmental improvements, and the inefficient subsidizing of subsistence farming, which enables the monopoly to gain legitimacy as a social provider. These dynamics underscore the complexities of Lebanon's tobacco supply chain and their importance for informed policymaking.
2. We Clearly defined the research question, ensuring it is concise and directly addresses the identified gap.
3. We articulated the unique contributions of the study, highlighting how it differs from and builds upon previous research as mentioned in point 1, as well as in our discussion of the importance of the qualitative approach adopted which offers a unique perspective that was not inspected in earlier studies on the tobacco supply chain in Lebanon. The qualitative approach is crucial as it provides an in-depth exploration of power dynamics among stakeholders in Lebanon's tobacco supply chain, which previous studies have overlooked. Additionally, it compensates for the lack of reliable quantitative data, offering valuable insights that are essential for understanding similar complex political economy relationships in other contexts.

A detailed overview of these changes is provided in our response to reviewer #2.

# Authors’ Response to Reviewer 1

The idea of the paper is interesting. There are concerns as follows: The rationale behind the chosen methodology with support from the academic literature.

**Response:** We thank the reviewer for their positive positive feedback. To address this concern, we have undertaken significant revisions to the manuscript to better explain the rationale behind our chosen methodology. In particular, we have amended the introduction, methodology and conclusion sections.

We now write in p. 9 *“According to Mathie and Camozzi (2005), qualitative research is especially useful for “politically or socially sensitive topics”, such as the dynamics of tobacco production and products smuggling. Furthermore, qualitative methods have been widely used in tobacco-related research. Newly published research delves into the governmental strategies and perspectives on tobacco control and its alternatives in Malawi. It involved semi-structured interviews with stakeholders working in the tobacco sector (Lencucha et al., 2020). Moreover, a recent study aimed to explore challenges facing tobacco control policies in Indonesia by conducting interviews with national tobacco control experts including academics, community organizations, and government officials (Astuti, Assunta, & Freeman, 2020). Another qualitative descriptive study in Australia focused on identifying key challenges hindering the progress in tobacco control by interviewing 31 individuals from various sectors involved in tobacco control. Similarly, a study aiming to understand the dynamics of the waterpipe industry also utilized semi-structured interviews with representatives from various waterpipe companies (Jongenelis, 2022). In many studies, the use of qualitative methodology has been considered a strength given the richness of data that it provides regarding the tobacco industry especially when official documents that could shed light on the market dynamics are lacking”.*

Going beyond support from the academic literature, we have also discussed the importance of the qualitative approach adopted which offers a unique perspective that was not inspected in earlier studies on the tobacco supply chain in Lebanon. The qualitative approach is crucial as it provides an in-depth exploration of power dynamics among stakeholders in Lebanon's tobacco supply chain, which previous studies have overlooked. Additionally, it compensates for the lack of reliable quantitative data, offering valuable insights that are essential for understanding similar complex political economy relationships in other contexts.

We now write in p.5 *“The adoption of a qualitative approach offers a unique perspective that was not inspected in earlier studies on the tobacco supply chain in Lebanon. First, it allows a detailed exploration of relationships of interests and power between different stakeholders (The monopole, farmers, private companies, regulators, and legislators). Second, given the limited availability and reliability of quantitative data on tobacco in Lebanon, and the deliberate ambiguity in public reports, the collected qualitative data offers crucial amount of information that would otherwise be unavailable. As these issues are not unique to the tobacco sector in Lebanon, the methodological approach adopted in this paper offers a blueprint to analysing political economy relationship in other context with similar structural complexities and data limitations.”*

The literature review section needs further attention. Papers worked on policy suggestions would be interesting to review.

For example:
Green subsidy modes and pricing strategy in a capital-constrained supply chain. Transportation Research Part E: Logistics and Transportation Review, 136, p.101885.
Alternative governmental carbon policies on populations of green and non-green supply chains in a competitive market. Environment, Development and Sustainability, 25(5), pp.4139-4172.
Joint impact of CSR policy and market structure on environmental sustainability in supply chains. Computers & Industrial Engineering, 185, p.109654.

This will, in turn, link the work to existing quantitative works in the literature that work on optimal policies. Such that will strengthen future work directions.

**Response:** We thank the reviewer for their suggestions regarding the inclusion of papers that focus on policy and would enhance the literature review section. While we highly appreciate the reviewer’s input, we have decided not to cite the specific works suggested, as we, along with the journal and editorial team, believe they are not directly related to our submitted manuscript. Instead, following the reviewer’s suggestion, we have cited recent policy-related papers that specifically address the most effective approaches for reducing tobacco smoking prevalence. In particular, we have added the following citations:

Burki S, et al. The Economics of Tobacco and Tobacco Taxation in Pakistan. Paris: International Union Against Tuberculosis and Lung Disease; 2013.

Foucade AL, et al. The potential for using alcohol and tobacco taxes to fund prevention and control of noncommunicable diseases in Caribbean Community countries. Revista panamericana de salud publica = Pan American journal of public health. 2018;42:e192-e. doi: 10.26633/RPSP.2018.192

Goodchild M, et al. Tobacco control and Healthy China 2030. Tobacco control. 2019;28(4):409-13. doi: 10.1136/tobaccocontrol-2018-054372

Levy DT, et al. Application of the Abridged SimSmoke model to four Eastern Mediterranean countries. Tobacco control. 2016;25(4):413-21. Epub 2015/06/16. doi: 10.1136/tobaccocontrol-2015-052334

Levy DT, et al. England SimSmoke: the impact of nicotine vaping on smoking prevalence and smoking-attributable deaths in England. Addiction (Abingdon, England). 2021;116(5):1196-211. Epub 20201008. doi: 10.1111/add.15269

Sánchez-Romero LM, et al. The Kentucky SimSmoke Tobacco Control Policy Model of Smokeless Tobacco and Cigarette Use. International journal of health policy and management. 2020. Epub 20201027. doi: 10.34172/ijhpm.2020.

Tesche J, et al. Measuring the effects of the new ECOWAS and WAEMU tobacco excise tax directives. Tobacco control. 2021;30(6):668-74. Epub 2020/09/30. doi: 10.1136/tobaccocontrol-2020-055843

Cho, A., Lim, C., Sun, T., Chan, G., & Gartner, C. (2024). The effect of tobacco tax increase on price‐minimizing tobacco purchasing behaviours: A systematic review and meta‐analysis. *Addiction*.

Additionally, and following reviewer # 2 suggestion, we have improved our introduction to cite more recent literature including that in 2024. In particular, we have added the following citations:

Soriano, J. B., Kendrick, P. J., Paulson, K. R., Gupta, V., Abrams, E. M., Adedoyin, R. A., ... & Moradi, M. (2020). Prevalence and attributable health burden of chronic respiratory diseases, 1990–2017: a systematic analysis for the Global Burden of Disease Study 2017. *The Lancet Respiratory Medicine*, *8*(6), 585-596.

Perez-Warnisher, M. T., de Miguel, M. P. C., & Seijo, L. M. (2018). Tobacco use worldwide: legislative efforts to curb consumption. *Annals of global health*, *84*(4), 571.

World Health Organization. (2024). **Tobacco use declines despite tobacco industry efforts to jeopardize progress.** Pan American Health Organization. Retrieved from [https://www.paho.org/en/news/16-1-2024-tobacco-use-declines-despite-tobacco-industry-efforts-jeopardize-progress](https://www.paho.org/en/news/16-1-2024-tobacco-use-declines-despite-tobacco-industry-efforts-jeopardize-progress).

Research and Markets. (2024). Global tobacco market report by type, and region 2024-2032. Retrieved from <https://www.researchandmarkets.com/reports/5993422/global-tobacco-market>.

El-Awa F, Bettcher D, Al-Lawati JA, Alebshehy R, Gouda H, Fraser CP. The status of tobacco control in the Eastern Mediterranean Region: progress in the implementation of the MPOWER measures. East Mediterr Health J. 2020 Jan 30;26(1):102-109. doi: 10.26719/2020.26.1.102. PMID: 32043552.

Saad RK, Maiteh A, Nakkash R, Salloum RG, Chalak A, Abu-Rmeileh NME, Khader Y, Al Nsour M. Monitoring and Combating Waterpipe Tobacco Smoking Through Surveillance and Taxation. JMIR Public Health Surveill. 2023 Mar 23;9:e40177. doi: 10.2196/40177. PMID: 36951907; PMCID: PMC10132023.

Robert West, Tobacco control: present and future, British Medical Bulletin, Volume 77-78, Issue 1, 2006, Pages 123–136, <https://doi.org/10.1093/bmb/ldl012>

U.S. National Cancer Institute and World Health Organization. The Economics of Tobacco and Tobacco Control. National Cancer Institute Tobacco Control Monograph 21. NIH Publication No. 16-CA-8029A. Bethesda, MD: U.S. Department of Health and Human Services, National Institutes of Health, National Cancer Institute; and Geneva, CH: World Health Organization; 2016.

Chaloupka, F. J., Yurekli, A., & Fong, G. T. (2012). Tobacco taxes as a tobacco control strategy. *Tobacco control*, *21*(2), 172-180.

Azagba, S., Ebling, T., & Korkmaz, A. (2024). Beyond the smoke: Historical analysis of the revenue implications of state cigarette tax policies, 1989 to 2019. *International Journal of Drug Policy*, *127*, 104408.

The conclusion section will need further revision accordingly.

**Response:** Following this round of revisions and considering the comments from the two anonymous reviewers, the conclusion of the manuscript has been substantially revised. We have addressed the feedback on the methodology, particularly by explaining the rationale behind it. Additionally, we have clarified the research question, objectives, and the gap our study addresses in the literature. We have also included the limitations and strengths of this study in the conclusion section, following the recommendation of Reviewer 2.

All modifications have been made in track changes mode.

# Authors’ Response to Reviewer 2

## Introduction

The introduction needs to be improved. Please, use more recent literature including those in 2024.

**Response:** We thank the reviewer for this suggestion. We have amended the introduction to incorporate more recent literature. Specifically, following reviewer 1's suggestion, we have cited recent policy-related papers that address the most effective approaches for reducing tobacco smoking prevalence. These new citations are listed in our response to reviewer 1's second comment and are not repeated here.

Following this suggestion, we have added the following citations:

- Soriano, J. B., Kendrick, P. J., Paulson, K. R., Gupta, V., Abrams, E. M., Adedoyin, R. A., ... & Moradi, M. (2020). Prevalence and attributable health burden of chronic respiratory diseases, 1990–2017: A systematic analysis for the Global Burden of Disease Study 2017. The Lancet Respiratory Medicine, 8(6), 585-596.

- Perez-Warnisher, M. T., de Miguel, M. P. C., & Seijo, L. M. (2018). Tobacco use worldwide: Legislative efforts to curb consumption. Annals of Global Health, 84(4), 571.

- World Health Organization. (2024). Tobacco use declines despite tobacco industry efforts to jeopardize progress. Pan American Health Organization. Retrieved from [https://www.paho.org/en/news/16-1-2024-tobacco-use-declines-despite-tobacco-industry-efforts-jeopardize-progress](https://www.paho.org/en/news/16-1-2024-tobacco-use-declines-despite-tobacco-industry-efforts-jeopardize-progress).

- Research and Markets. (2024). Global tobacco market report by type, and region 2024-2032. Retrieved from [https://www.researchandmarkets.com/reports/5993422/global-tobacco-market](https://www.researchandmarkets.com/reports/5993422/global-tobacco-market).

- El-Awa, F., Bettcher, D., Al-Lawati, J. A., Alebshehy, R., Gouda, H., & Fraser, C. P. (2020). The status of tobacco control in the Eastern Mediterranean Region: Progress in the implementation of the MPOWER measures. Eastern Mediterranean Health Journal, 26(1), 102-109. doi: 10.26719/2020.26.1.102. PMID: 32043552.

- Saad, R. K., Maiteh, A., Nakkash, R., Salloum, R. G., Chalak, A., Abu-Rmeileh, N. M. E., & Khader, Y. (2023). Monitoring and combating waterpipe tobacco smoking through surveillance and taxation. JMIR Public Health and Surveillance, 9, e40177. doi: 10.2196/40177. PMID: 36951907; PMCID: PMC10132023.

- West, R. (2023). Tobacco control: Present and future. British Medical Bulletin, 77(1), 123–136. doi: 10.1093/bmb/ldl012.

- U.S. National Cancer Institute and World Health Organization. (2016). The economics of tobacco and tobacco control. National Cancer Institute Tobacco Control Monograph 21. NIH Publication No. 16-CA-8029A. Bethesda, MD: U.S. Department of Health and Human Services, National Institutes of Health, National Cancer Institute; and Geneva, CH: World Health Organization.

- Chaloupka, F. J., Yurekli, A., & Fong, G. T. (2012). Tobacco taxes as a tobacco control strategy. Tobacco Control, 21(2), 172-180.

- Azagba, S., Ebling, T., & Korkmaz, A. (2024). Beyond the smoke: Historical analysis of the revenue implications of state cigarette tax policies, 1989 to 2019. International Journal of Drug Policy, 127, 104408.

The problem or gap is not well argued out. You must properly pitch your study in extant literature.

**Response:**

SDSD

**Response:** Thank you for this comment. We acknowledge your concern regarding the need to better articulate the problem or gap in our study and to position our research within the existing literature.

In response, we have revised the introduction and literature review sections to more clearly define the research gap and to situate our study within the current body of knowledge. Specifically, we have:

1. Enhanced our discussion of the existing literature to highlight the areas where our research contributes new insights.
2. Clearly articulated the specific problem we are addressing and the significance of this issue in the broader context.
3. Strengthened our argument by providing more detailed evidence and examples from relevant studies to support the necessity and relevance of our research.

Here is a list of modifications that support points 1 through 3:

We now write in p. 4 ***“The core question that this paper explores is how does the prevalent market structure impact the influence and relative power of the different stakeholders in the tobacco market.”***

We now write in p.5 *“The adoption of a qualitative approach offers a unique perspective that was not inspected in earlier studies on the tobacco supply chain in Lebanon. First, it allows a detailed exploration of relationships of interests and power between different stakeholders (The monopole, farmers, private companies, regulators, and legislators). Second, given the limited availability and reliability of quantitative data on tobacco in Lebanon, and the deliberate ambiguity in public reports, the collected qualitative data offers crucial amount of information that would otherwise be unavailable. As these issues are not unique to the tobacco sector in Lebanon, the methodological approach adopted in this paper offers a blueprint to analysing political economy relationship in other context with similar structural complexities and data limitations.”*

We now write in p. 6 and 7 *“This study complements and add to this body of literature, by highlighting two significant issues: 1) the misalignment of policy objective between the monopole, with a profit maximizing objective, and the public policy makers who target several policy objectives, including maximizing of net public revenues, improvement of public health and environmental objectives; 2) the co-dependency between monopole and subsistence farming, by subsidizing and encouraging an inefficient system of substance farming, the monopole attain legitimacy as a social provider. Overall, this study offers a comprehensive picture of the complex relationships in the tobacco supply chain in Lebanon, which should serve as a chart for any feasible and efficient policymaking.”*

All modifications have been made in track changes mode.

Besides, it is not enough to say “…far less attention has been dedicated to examining the political economy of tobacco supply chains and associated, tobacco industry practices, as important as such an understanding is to the design and implementation of effective taxation policies.” And so what? What is the effect of the above? You must make a good case for your study.

**Response:** Thank you for your thoughtful comment, which highlights the importance of establishing the significance of our study, clarifying the research question, and distinguishing our contributions from existing work. The response we provided to earlier feedback from reviewers 1 and 2 addresses this concern.

To avoid repetition, we have not included our recently added sections that discuss the study's importance, contributions, and research focus. In summary, our goal is to examine how the current market structure shapes the influence and power dynamics among various stakeholders in the tobacco industry, which is crucial for crafting effective tax policies. An in-depth analysis of the interactions and power relationships among key players—such as the monopoly, farmers, private companies, regulators, and legislators—is crucial for understanding Lebanon's tobacco sector. The study identifies two primary issues in Lebanon: first, the conflict between the profit-driven goals of the monopoly and the broader objectives of public officials, such as enhancing public revenue, health, and environmental outcomes; and second, the reliance between the monopoly and subsistence farming, where support for inefficient farming practices allows the monopoly to establish itself as a social provider. By identifying these two issues and by understanding the relationships within Lebanon's tobacco supply chain, this study provides valuable insights for developing effective policies.

Why have “background?” I suggest you find a way to clearly merge the background with the introduction.

**Response:** We thank the reviewer for this suggestion. We have now amended the introduction to integrate the background section.

We have incorporated part of the background section content as a footnote (#1) in the introduction, specifically regarding the size of Lebanon’s tobacco leaf cultivation industry. Additionally, we have included the historical background on the structure of the tobacco supply chain in Lebanon as a concluding paragraph in the introduction, right before the paragraph that explains the sections and organization of the manuscript.

All modifications have been made in track changes mode.

Please condense your objective into a research question.

**Response:** Thank you for your valuable suggestion. We have amended the manuscript to clearly state our objective and articulate it into a research question. We now write in the manuscript p. 4 *“****The core question that this paper explores is how does the prevalent market structure impact the influence and relative power of the different stakeholders in the tobacco market.”***

All modifications have been made in track changes mode.

You must also clearly state the contributions of your study….unlike previous studies.

**Response:** We thank the reviewer for this suggestion. We believe we have addressed the reviewer’s concern by making significant changes to the manuscript. The revised version now clearly clarifies the research gap, question, objectives, and contributions.

We now write in p.5 *“The adoption of a qualitative approach offers a unique perspective that was not inspected in earlier studies on the tobacco supply chain in Lebanon. First, it allows a detailed exploration of relationships of interests and power between different stakeholders (The monopole, farmers, private companies, regulators, and legislators). Second, given the limited availability and reliability of quantitative data on tobacco in Lebanon, and the deliberate ambiguity in public reports, the collected qualitative data offers crucial amount of information that would otherwise be unavailable. As these issues are not unique to the tobacco sector in Lebanon, the methodological approach adopted in this paper offers a blueprint to analysing political economy relationship in other context with similar structural complexities and data limitations.”*

We now write in p. 6 and 7 *“This study complements and add to this body of literature, by highlighting two significant issues: 1) the misalignment of policy objective between the monopole, with a profit maximizing objective, and the public policy makers who target several policy objectives, including maximizing of net public revenues, improvement of public health and environmental objectives; 2) the co-dependency between monopole and subsistence farming, by subsidizing and encouraging an inefficient system of substance farming, the monopole attain legitimacy as a social provider. Overall, this study offers a comprehensive picture of the complex relationships in the tobacco supply chain in Lebanon, which should serve as a chart for any feasible and efficient policymaking.”*

All modifications have been made in track changes mode.

Don’t make the introduction too long…it makes the reading boring.

**Response:** Thank you for your feedback. We understand the concern about the length of the introduction and its potential impact on reader engagement. In response to various comments from two anonymous reviewers, we have made comprehensive changes throughout the manuscript, including the introduction. While these revisions required us to maintain the detailed content, we have reorganized the flow to make it more cohesive and engaging for the reader. We believe these adjustments enhance the readability and clarity of the introduction.

All modifications have been made in track changes mode.

## Methodology

Why do you have “methodology and sample description”? isn’t sample description part of the methodology? Refer to the structure of similar papers in this journal and properly label your captions under the methodology.

**Response:** We thank the reviewer for this suggestion. The sample description is indeed part of the methodology, similar to the section titled “3.2 Population, Sample and Sampling Technique of the Study” in Kwarteng et al. (2022). We understand the concern about the titles of the paragraphs under the methodology section. To address this, we have reorganized the subsections as follows:

Under the methodology and sample description, we have retained the following subsections: Study Design; Population of Interest and Sample Size; Recruitment and Data Collection; Data Management and Analysis; and Ethical Considerations.

We have incorporated the content of the previous subsections (from the originally submitted manuscript) regarding the validation workshop under "Population of Interest and Sample Size". Additionally, we have included paragraphs related to the interview approach and methods to improve rigor under the "Recruitment and Data Collection" section, as outlined in Kwarteng et al. (2022). Lastly, we have detailed the thematic analysis under "Data Management and Analysis".

We hope that these adjustments address the concern.

You must justify the choice of each technique. For example, why you used qualitative or purposive sampling?

**Response:** We thank the reviewer for this recommendation. The methodology section now includes a detailed explanation about the rationale behind the use of each technique.

First, as mentioned in our reply to reviewer 1’s first comment, the use of qualitative research has been supported by evidence from the literature. It is considered as a strength in the context of tobacco research given the depth and richness of data which it provides when compared to quantitative methodologies.

In our response to reviewer 1’s first comment, we have added the following paragraph to the manuscript in p.9: *“According to Mathie and Camozzi (2005), qualitative research is especially useful for “politically or socially sensitive topics”, such as the dynamics of tobacco production and products smuggling. Furthermore, qualitative methods have been widely used in tobacco-related research. Newly published research delves into the governmental strategies and perspectives on tobacco control and its alternatives in Malawi. It involved semi-structured interviews with stakeholders working in the tobacco sector (Lencucha et al., 2020). Moreover, a recent study aimed to explore challenges facing tobacco control policies in Indonesia by conducting interviews with national tobacco control experts including academics, community organisations, and government officials (Astuti, Assunta, & Freeman, 2020). Another qualitative descriptive study in Australia focused on identifying key challenges hindering the progress in tobacco control by interviewing 31 individuals from various sectors involved in tobacco control. Similarly, a study aiming to understand the dynamics of the waterpipe industry also utilized semi-structured interviews with representatives from various waterpipe companies (Jongenelis, 2022). In many studies, the use of qualitative methodology has been considered a strength given the richness of data that it provides regarding the tobacco industry especially when official documents that could shed light on the market dynamics are lacking”.*

Second, purposive sampling has been chosen to recruit stakeholders of interest which are people involved in the tobacco sector including policy advocates, researchers, and government officials.

We now write in p. 11: *“Purposive sampling was employed to select and recruit stakeholders based on their professional roles and direct involvement in this area, and to optimize the use of research resources”.*

Third, we defined and justified the use of thematic analysis in the context of this particular study.

We now write in p. 12: *“Thematic analysis is “a method for identifying, analyzing, and interpreting patterns of meaning (‘themes’) within qualitative data” (Clarke & Braun, 2017, p.297-298). It has been used since it is flexible and can be adapted to our research question and sample size. It is also systematic, generates rigorous analysis, and provides interpretive depth (Clarke & Braun, 2017)”.*

Fourth, we justified the use of the inductive analysis approach to emphasize that findings were data-driven. We now write in p.12: *“Data analysis has been conducted concurrently with ongoing data collection and adopted an inductive coding approach. This approach was used to extract themes from raw data, enabling the identification of emerging topics relevant to the research question and the discovery of new, unexpected themes without any preconceptions or guiding hypotheses (Bingham & Witkowsky, 2021)”.*

How was the analysis done? The present status is too shallow. Did you use software or manual? And how the themes discovered? Did you do immersion? Coding? Refer to Kwarteng et al., (2022) for more insight into thematic analysis. This part of the work is weak. This important to guarantee transparency and trust in your results.

**Response:** We thank the reviewer for this suggestion. We have provided a more detailed explanation of our data analysis and have outlined the steps taken in the thematic analysis including immersion, coding, and themes generation.

We now write in p.12-13: “*Data analysis has been conducted concurrently with ongoing data collection and adopted an inductive coding approach. This approach was used to extract themes from raw data, enabling the identification of emerging topics relevant to the research question and the discovery of new, unexpected themes without any preconceptions or guiding hypotheses (Bingham & Witkowsky, 2021). The analysis was conducted manually using a thematic approach described by Braun and Clarke (2006) [23]. Thematic analysis is “a method for identifying, analyzing, and interpreting patterns of meaning (‘themes’) within qualitative data” (Clarke & Braun, 2017, p.297-298). It has been used since it is flexible and can be adapted to our research question and sample size. It is also systematic, generates rigorous analysis, and provides interpretive depth (Clarke & Braun, 2017). It consists of six phases, starting from the immersion in the data, generating initial codes by systematically coding across the data set, identifying common themes and grouping codes into themes, revising the themes and ensuring coherence, refining and naming the themes, until reaching the final step which includes embedding the themes in an analytic tale and writing the report. Findings and emergent themes were supported with quotes from individual interviews”.*

Also refer to Kanda et al. (2021) and Kwarteng et al., (2022) to see how they presented their results. Use insights from these papers to better your presentation of results.

**Response:** We thank the reviewer for this suggestion. We now write in p. 13-14: *“In the course of data analysis, five main themes emerged, shedding light on the dynamics and landscape of the tobacco industry in Lebanon as follows: The Regie: a bilateral monopoly, Tobacco farming: challenges and dynamics, big tobacco companies, distribution and wholesale, public oversight and public policy”.*

Some aspects of the discussions were done from the perspective of extant literature (What we already know) that is good. This must be replicated through the discussions (where applicable).

**Response:** We appreciate the reviewer's suggestion. We have revised the discussion and policy recommendations sections to better integrate them with existing literature, providing relevant citations where applicable. This includes incorporating references to studies outside our specific context that support our arguments, thereby enhancing the external validity and generalizability of our findings.

Particularly, we have added the following list of citations:

- Chaaban, J., Salti, N., Naamani, N., et al. (2019). "The economics of tobacco in Lebanon: an estimation of the social costs of tobacco consumption." *Tobacco Control*, 28(4), 418-423.

- Chaloupka, F. J., Yurekli, A., & Fong, G. T. (2012). "Tobacco taxes as a tobacco control strategy." *Tobacco Control*, 21(2), 172-180.

- Jha, P., & Peto, R. (2014). "Global effects of smoking, of quitting, and of taxing tobacco." *New England Journal of Medicine*, 370(1), 60-68.

- Fuchs, A., & Meneses, F. (2017). "Tobacco price elasticity and tax progressivity in Moldova." *World Bank Economic Review*, 31(3), 634-652.

- Goodchild, M., Perucic, A. M., & Nargis, N. (2016). "Modelling the impact of raising tobacco taxes on public health and finance." *Bulletin of the World Health Organization*, 94(4), 250-257.

- Laverty, A. A., Li, C. R., Chang, K. C., Millett, C., & Filippidis, F. T. (2023). Cigarette taxation and price differentials in 195 countries during 2014–2018. *Tobacco Control*, *32*(3), 359-365.

- Vardavas, C. I., Filippidis, F. T., Agaku, I. T., Mytaras, V., Bertic, M., Connolly, G. N. et al. (2012). Tobacco taxation: the importance of earmarking the revenue to health care and tobacco control. Tobacco Induced Diseases, 10(December), 21. https://doi.org/10.1186/1617-9625-10-21

- Igoumenidis, M., & Athanasakis, K. (2014). Earmarking Tobacco Taxes for Health Purposes via Median Entities. International Journal of Health Policy and Management, 3(3), 159-159. doi: 10.15171/ijhpm.2014.72

I discovered that there was no theoretical background in the study. Neither were the discuses done in line with any specific theory. Anyway, is the study an inductive or deductive one? Don’t you think it would be nice to mention how your study supports or disprove some theories. OR if you are doing an inductive research, we must state in your contribution how you are contributing to the development of theory. This is seriously lacking in your study.

**Response:** We thank the reviewer for raising this concern. We have revised the manuscript to provide a more thorough discussion of the inductive research approach and its potential theoretical contribution.

We now write in p. 7 *“This study employs an inductive approach which is motivated by the exploratory nature of the investigation into the political economy of the tobacco supply chain in Lebanon. Inductive research, as undertaken here, begins with specific observations and aims to lead to broader policy and conceptual insights. This approach is particularly suitable for understanding the complex but theoretically under-explored context like the tobacco industry and supply chain in Lebanon, where empirical data is scarce, and the relationships among stakeholders are complex and yet poorly understood. This study therefore attempts to propose a nuanced understanding of the interplay between state-owned monopolies, subsistence farming, and market dynamics within a politically sensitive industry.”*

## Conclusion

The conclusion must be well written. It must capture the gap, research question, methods and summary of the findings

**Response:** We appreciate the reviewer’s comment. In response, we have significantly revised the conclusion to address various feedback, including improving the articulation of the research question, identifying the research gap, and clarifying the study's contribution, methodology, and findings. Additionally, we have emphasized the strengths and limitations, which are discussed in detail in the following points.

All modifications have been made in track changes mode.

Also provide the theoretical and practical contribution of study

**Response:** Thank you for your comment. We have now amended the conclusion to emphasize on the the study's theoretical and practical contributions.

All modifications have been made in track changes mode.

What are the limitation and directions for future studies?

**Response:** We appreciate the reviewer for bringing this to our attention. We would like to note that the manuscript already includes a discussion of the limitations, strengths, and directions for future research in the conclusion section. However, following the reviewer’s suggestion, we have now organized these points as a subtopic under the conclusion to enhance clarity and readability for the reader.

Each of the above (2 and 3) should be subtopics under the conclusion

**Response:** Following the reviewer's advice, we have reorganized the conclusion to emphasize the study's strengths and limitations, which are now presented as a subtopic. For coherence, we have integrated the discussion of theoretical and practical contributions throughout the conclusion rather than separating them into a distinct subtopic.

**Response:** We thank the reviewer for this valuable feedback. In response, we have used AI tools to improve the language quality of our manuscript, enhancing the grammar, clarity, and overall readability of the text.

The language must be improved. Get a language expert to check the grammar.

**Response:**

Ensure that all citations are properly referenced.

**Response:**

**Response:** Thank you for this comment. We have carefully reviewed the manuscript to ensure that all citations are properly referenced and consistently formatted throughout the text. All modifications to references are in track changes.
