# Supplementary material for: A political economy of the tobacco supply chain in an Eastern Mediterranean country: The case of Lebanon
Source: PLoS One. 2025 Mar 31;20(3):e0320050. doi: 10.1371/journal.pone.0320050 (PMC11957285; doi:10.1371/journal.pone.0320050)
Supplement: Attachment — Submitted filename: Reviewer comments (Political Economy).docx [file pone.0320050.s002.docx]

Reviewer comments

Introduction

1. The introduction needs to be improved. Please, use more recent literature including those in 2024.
2. The problem or gap is not well argued out. You must properly pitch your study in extant literature.
3. Besides, it is not enough to say “…far less attention has been dedicated to examining the political economy of tobacco supply chains and associated, tobacco industry practices, as important as such an understanding is to the design and implementation of effective taxation policies.” And so what? What is the effect of the above? You must make a good case for your study.
4. Why have “background?” I suggest you find a way to clearly merge the background with the introduction.
5. Please condense your objective into a research question.
6. You must also clearly state the contributions of your study….unlike previous studies.
7. Don’t make the introduction too long…it makes the reading boring.

Methodology

1. Why do you have “methodology and sample description” ? isn’t sample description part of the methodology? Refer to the structure of similar papers in this journal and properly label your captions under the methodology.
2. You must justify the choice of each technique. For example, why you used qualitative or purposive sampling?
3. How was the analysis done? The present status is too shallow. Did you use software or manual? And how the themes discovered? Did you do immersion? Coding? Refer to Kwarteng et al., (2022) for more insight into thematic analysis. This part of the work is weak. This important to guarantee transparency and trust in your results.
4. Also refer to Kanda et al. (2021) and Kwarteng et al., (2022) to see how they presented their results. Use insights from these papers to better your presentation of results.
5. Some aspects of the discussions were done from the perspective of extant literature (What we already know) that is good. This must be replicated through the discussions (where applicable).
6. I discovered that there was no theoretical background in the study. Neither were the discuses done in line with any specific theory. Anyway, is the study an inductive or deductive one? Don’t you think it would be nice to mention how your study supports or disprove some theories. OR if you are doing an inductive research, we must state in your contribution how you are contributing to the development of theory. This is seriously lacking in your study.

Conclusion

1. The conclusion must be well written. It must capture the gap, research question, methods and summary of the findings
2. Also provide the theoretical and practical contribution of study
3. What are the limitation and directions for future studies?
4. Each of the above (2 and 3) should be subtopics under the conclusion
5. The language must be improved. Get a language expert to check the grammar
6. Ensure that all citations are properly referenced.
